# Drug like HSP27 cross linkers with chromenone structure ameliorates pulmonary fibrosis

**DOI:** 10.3389/fphar.2023.1203033

**Published:** 2023-07-04

**Authors:** Young Jo Yoo, Seulgi Jeon, Hee Jin, Hee Yeon Won, Mi Gyeong Jeong, Yeseul Cho, Eun Sook Hwang, Younghwa Na, Jaeho Cho, Yun-Sil Lee

**Affiliations:** ^1^ Graduate School of Pharmaceutical Sciences, Ewha Womans University, Seoul, Republic of Korea; ^2^ College of Pharmacy, CHA University, Pocheon-si, Gyeonggi-do, Republic of Korea; ^3^ Department of Radiation Oncology, Yonsei University Health System, Seoul, Republic of Korea

**Keywords:** heat shock protein 27, cross linking inhibitors, NA49, pulmonary fibrosis, radiation, bleomycin

## Abstract

**Background:** Pulmonary fibrosis (PF) is a progressive lung disease characterized by fibroblast accumulation and collagen deposition, resulting in lung scarring and impaired gas exchange. Current treatments for idiopathic pulmonary fibrosis (IPF) have limited efficacy and significant side effects. Heat shock protein 27 (HSP27) has emerged as a potential therapeutic target for PF due to its involvement in fibrotic processes. However, effective HSP27 inhibitors for PF treatment are still lacking.

**Methods:** To assess the anti-fibrotic effects of NA49, we utilized murine PF models induced by radiation (IR) or bleomycin (BLM). We administered NA49 to the PF mice and evaluated its impact on lung fibrosis progression. We also investigated the molecular mechanisms underlying NA49's effects, focusing on its inhibition of EMT-related signaling pathways.

**Results:** In our study, we evaluated the potential of a novel HSP27 inhibitor, NA49, in preclinical models of PF. NA49 effectively suppressed PF development in radiation and bleomycin-induced PF models. It reduced fibrosis, inhibited NFkB signaling, and downregulated EMT-related molecules. Importantly, we evaluated the safety profile of NA49 by assessing its impact on DNA strand breakage. Compared to previous HSP27 inhibitors, NA49 showed lower levels of DNA damage in human lung epithelial cells, and suggests that NA49 may have reduced toxicity compared to other HSP27 inhibitors. Overall, our results demonstrate that NA49 effectively inhibits PF development in preclinical models. It reduces lung fibrosis, inhibits EMT-related signaling pathways, and exhibits improved safety profiles. These findings highlight the potential of NA49 as a promising candidate for the treatment of PF.

**Conclusion:** NA49 exhibited significant anti-fibrotic effects, inhibiting fibrosis development and EMT-related signaling pathways. Moreover, NA49 showed improved safety profiles compared to previous HSP27 inhibitors.

## Introduction

Pulmonary fibrosis (PF), which includes idiopathic pulmonary fibrosis (IPF), primarily affects older individuals, with a typical age of diagnosis in the mid-60s (Raghu et al., 2006). The underlying mechanism of PF is believed to involve repeated micro injuries to the alveolar epithelium and a severely disrupted injury-repair response. This leads to the accumulation of fibroblasts and collagen, resulting in lung scarring and impaired gas exchange (Wilson and Wynn, 2009). The prevailing theory is that fibrosis in PF follows a pathway comparable to the normal process of wound healing (Wynn, 2011). Instead of concluding the reparative process with the elimination of fibroblasts during the maturation phase, a continuous fibrotic response ensues, sustained by a positive feedback mechanism involving the proliferation and activation of fibroblasts, production of extracellular matrix (ECM), and inhibition of fibroblast apoptosis (Wynn, 2011; White and Mantovani, 2013).

Three pharmacological treatments for IPF, pirfenidone (PFD), nintedanib (NTD), and N-acetylcysteine (NAC) are currently commercially available. PFD, by inhibiting TGFβ, has demonstrated its ability to prevent the accumulation of hydroxyproline, procollagen I and III, inflammatory cells, and TGFβ1 in bronchoalveolar lavage and/or lung tissue (Westergren-Thorsson et al., 1993; Iyer et al., 1999a; Iyer et al., 1999b; Oku et al., 2008; Carter, 2011; Myllärniemi and Kaarteenaho, 2015). In mouse models of PF, PFD has exhibited the ability to reduce the population of fibrocytes and inhibit their migration (Inomata et al., 2014). Nintedanib (NTD), a pan-tyrosine kinase receptor inhibitor, was serendipitously discovered as a byproduct during extensive screening assays originally intended for targeting cyclin-dependent kinase (Roth et al., 2015). However, conclusive evidence establishing its clinical efficacy in the treatment of PF is currently lacking. Although these drugs have shown effectiveness in slowing the decline in lung function and reducing the risk of acute respiratory deterioration, they are associated with a substantial occurrence of morbidity and mortality. Notably, individual clinical trials have not demonstrated a reduction in mortality (Roth et al., 2015). Furthermore, both therapies commonly exhibit side effects, primarily affecting the gastrointestinal system (Richeldi et al., 2014).

Heat shock protein 27 (HSP27), known as HSP27 in humans and HSP25 in mice, is an ATP-independent molecular chaperone that exhibits significant upregulation in response to various cellular stresses (Ferns et al., 2006). HSP27 promotes the migration and invasion of cancer cells and facilitates the process of epithelial-to-mesenchymal transition (EMT) (Shiota et al., 2013). It also induces EMT during fibrosis, including IPF (Hill et al., 2019). HSP27 overexpression has been reported in patients diagnosed with IPF (Park et al., 2016). Elevated levels of HSP27 play a significant role in the development of myofibroblasts and may serve as a potential therapeutic target for fibrotic disorders. The suppression of the HSP27 gene using OGX-427, a second-generation antisense oligonucleotide, has been shown to decrease PF induced by bleomycin (BLM) and inhibit EMT through the degradation of Snail (Wettstein et al., 2013). In addition, we identified HPS27 as a molecular target for PF and functional inhibition of HSP27 using a small molecule J2, a HSP27 cross-linker (Choi et al., 2017; Hwang et al., 2017), ameliorated PF. The activation of IkBα-NFκB signaling, facilitated by the direct interaction between IkBα and HSP27, plays a crucial role in the process of EMT, which is closely associated with the development of PF (Kim et al., 2019). As a consequence, HSP27 inhibition is an appealing treatment option, even though HSP27, unlike HSP90 and HSP70, lacks an ATP-binding site, thus to date, no effective inhibitors targeting HSP27 have been identified.

Previously, we have been working on the development of different HSP27 inhibitors that induce cross-linking of HSP27 by inserting themselves between the disulfide bonds of HSP27. Some examples of these inhibitors include zerumbone, which was derived from a natural product, SW15, a synthetic xanthone compound (Kim et al., 2016), and J2, a chromenone compound (Choi et al., 2017; Hwang et al., 2017) However, these compounds faced the challenges in terms of their poor drugability or toxicity. Therefore, we developed NA49, a J2 derivative, which showed better drugability in absorption, distribution, metabolism, excretion, and toxicity (ADME/Tox) (Yoo et al., 2021).

In the current study, we investigated the potential of NA49 as a more effective HSP27 inhibitor for the treatment of PF. To assess its antifibrotic effects, we used murine PF models induced by radiation (IR) or BLM and NA49 may be a good drug candidate for overcoming PF by blocking the EMT stage.

## Materials and methods

For a comprehensive description of the Materials and methods, please refer to the Supplementary Material.

### Chemicals

J2 (Choi et al., 2017; Hwang et al., 2017) and NA49 (Yoo et al., 2021) were chemically synthesized and previously described.

### IR and BLM induced PF models

All experimental procedures were granted approval by the Animal Care and Use Committees of Ewha Womans University (IACUC 18–064) and Yonsei University Medical School (2015–0267) and were conducted in strict adherence to the applicable guidelines. C57BL/6N mice (male, 6 weeks old, with a minimum of three mice per group) were obtained from JOA bio Inc. (Seoul, Korea). The mice were either irradiated with a single dose of 75 Gy using the X-RAD 320 platform as described previously (Jin et al., 2017) or received a single intratracheal instillation of 2.5 mg/kg BLM (Santacruz Biotechnology, Dallas, TX, United States). NA49 and J2 (3 mg/kg) were intraperitoneally injected every other day for 6 weeks or 2 weeks after IR or BLM treatment, respectively. C57BL/6N wild-type (WT) mice and HSP25 transgenic (TG) mice were generated and housed under pathogen-free conditions at Macrogen, Inc. (Kim et al., 2019).

### Cell culture and immunoblotting

The L132 cell line (human normal lung epithelial cell line) was obtained from the ATCC (RPMI media) and the KCLB (Korean Cell Lines Bank) (DMEM media). The cells were cultured in media (Gibco) supplemented with 10% FBS (Gibco) and maintained in a 37°C incubator with 5% CO_2_. To ensure the absence of *Mycoplasma* contamination, the cell lines were tested using the BioMycoX *Mycoplasma* PCR Detection Kit (JCBIO Co., Ltd.). The L132 cells were extracted using RIPA lysis buffer, and the cell extracts were subjected to SDS-polyacrylamide gel electrophoresis under reducing conditions and subsequently transferred onto a nitrocellulose membrane. After incubation with primary antibodies, the protein blot was next probed with the appropriate secondary antibodies that had been conjugated to HRP. The ECL detection technology was used in order to see the protein bands (GE Healthcare, Freiburg, Germany). At least three separate immunoblots were used to provide the quantitative protein analysis that was achieved. After being normalized to β-actin, the quantification of the detected intensities of the protein bands was then performed using the ImageJ program. The antibodies that were tested for this investigation may be found listed in Supplementary Table S1.

### Quantitative RT-PCR

Total RNA was extracted from the cells or tissues using TRIzol reagent (Qiazen, Valencia, CA, United States). A Nanodrop was used to evaluate RNA purity and concentration. According to the manufacturer’s instructions, RNA was reverse transcribed using the ReverTra Ace^®^ qPCR RT Kit (TOYOBO, Osaka, Japan). For quantitative RT-PCR, the THUNDERBIRD SYBR qPCR Mix (TOYOBO, Osaka, Japan), and the Step One Plus RT-PCR machine (Applied Biosystems, Carlsbad, CA, United States) were used. After normalization using the Ct values of the GAPDH gene, the relative transcript levels of genes were determined. The primers used are detailed in Supplementary Table S2.

### Preparation of lung tissues for immunohistochemistry and immunofluorescence staining

Lung tissue was removed from IR or BLM-treated mice, fixed in 10% (v/v) neutral buffered formalin and paraffin embedding was performed to prepare the samples. The paraffin-embedded sections were deparaffinized and subsequently stained using hematoxylin and eosin (H&E, Sigma-Aldrich) as well as a Masson’s trichrome (MT) staining kit (Sigma-Aldrich). Additionally, immunohistochemistry, and immunofluorescence staining were carried out. Images were acquired using a Zeiss Apotome microscope (Carl Zeiss) installed at the Ewha Drug Development Research Core Center. All antibodies used in the study were listed in Supplementary Table S3.

### Irradiation

The cells in this study were subjected to IR with a single dose of either 5 or 10 Gy. The IR was generated using a ^137^Cs gamma-ray source (Elan 3000, Atomic Energy of Canada, Mississauga, Canada) at a dose rate of 3.81 Gy/min. To ensure radiation safety, radiation workers underwent annual radiation safety management training conducted by the Korea Foundation of Nuclear Safety (KoFONS).

### Micro-CT analysis

Micro-CT analysis was performed as described previously (Kim et al., 2017). High-resolution micro-computed tomography (CT) images were obtained using a volumetric CT scanner (NFR Polaris- G90MVC: NanoFocusRay, Iksan, South Korea) operating at 50 kVp, 180 μA, and 150 mGy. The scanning parameters included 700 views and a frame rate of 142 ms. The images were reconstructed using volumetric cone-beam reconstruction with the Feldkamp-Davis-Kress method. The reconstructed images had a size of 1,232 × 1,120 pixels and consisted of 512 slices. For the analysis, ImageJ software was utilized for volumetric measurements. To ensure consistent measurements across specimens, the same level settings were applied to all images during the analysis.

### Alkaline comet assay

At 37°C, 1 × 10^5^ cells/mL L132 suspended in PBS were combined with low-melting agarose in a 1:10 (v/v) ratio. The combination was applied to a CometSlide (Trevigen Inc., Maryland, United States, #4250-200-03). After the agarose had solidified, the slides were submerged in a lysis solution for 1 h. They were then placed in an alkaline unwinding solution comprising 200 mM NaOH and 200 mM EDTA (pH 13) and incubated at room temperature for 20 min. At 4°C, an electrical field (25 V) was administered for 30 min. After being stored at 4°C for 1 h to overnight, the slides were stained with 70 μL of ×1 SYBR Gold (Thermo Fisher Scientific, #S11494) and the comet photos were acquired using a Zeiss confocal microscope. The Komet 5.5 software application was used to examine the datasets.

### Statistical analysis

One-way ANOVA was used to determine the statistical significance among and between conditions by Tukey’s test or using GraphPad Prism Software version 9.0. All data were obtained from a minimum of three independent experiments. Data are reported as the mean ± SEM.

## Results

### Inhibition of EMT by NA49, a druggable HSP27 cross-linker

**FIGURE 1:**
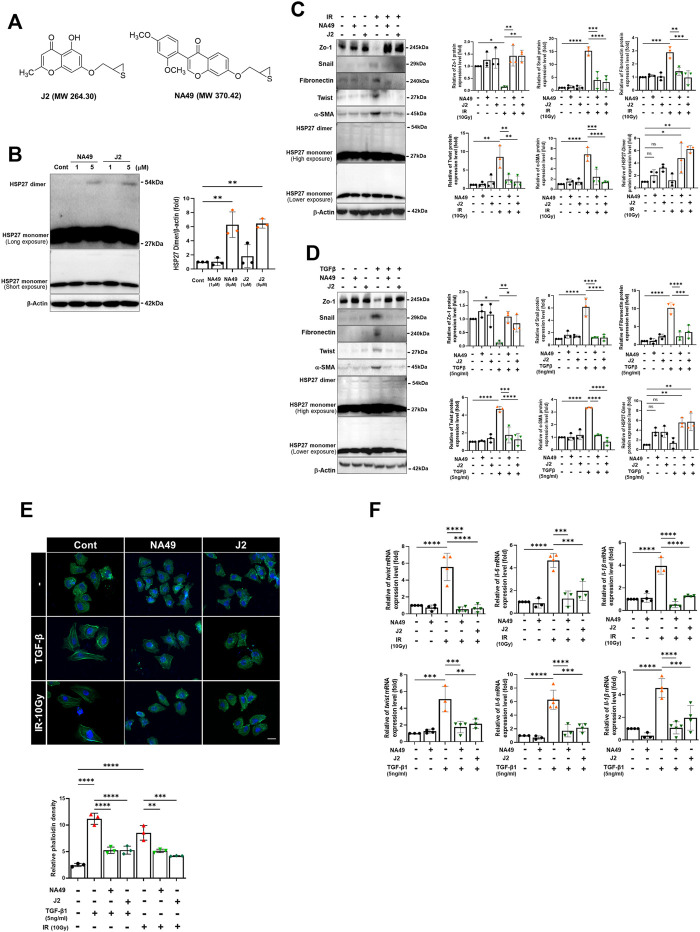
HSP27 cross-linker NA49 inhibits EMT in lung cell lines. **(A)** Chemical structures of NA49 and J2. **(B)** The protein expression levels after treatment of HSP27 cross-linkers NA49 and J2 (1 or 5 µM) for 9 h were verified by immunoblot analysis. The intensity band was quantified by ImageJ software, and the data were normalized to β-actin. **(C,D)** Immunoblot analysis using cell lysates of L132 cells at 24 and 48 h of 10 Gy irradiation (IR) or 5 ng/mL TGFβ treatment with or without NA49 and J2 (0.5 µM) pretreatment were performed. The intensity band was quantified by ImageJ software, and the data were normalized to β-actin. **(E)** L132 cells were pre-treated with 0.5 µM NA49 or J2 for 2 h and then exposed to IR of 10 Gy. At 48 h post-irradiation, the levels of phalloidin (green) and DAPI (blue) were evaluated using immunofluorescence staining. Magnification at ×400. **(F)** Cell lysate of L132 cells were irradiated with 10 Gy IR after pretreatment of NA49 or J2 (0.5 µM) and after 24 h, quantitative RT-PCR analysis was performed. Data are expressed as mean ±SEM; Scale bar: Magnification, ×400; Scale bar: 50 µm *p < 0.05 **p < 0.01 ***p < 0.001 and ****p < 0.0001 (p-valued by ANOVA).

A previous study demonstrated that J2 induces the cross-linking of HSP27, leading to the formation of altered dimers. These altered dimers result in the functional inhibition of HSP27, ultimately leading to the inhibition of PF (Kim et al., 2019). However, due to the inadequate solubility and short *in vivo* circulation time of J2, we have developed a more druggable compound, NA49 (Figure 1A). In comparison to J2, NA49 exhibited a superior pharmacokinetic profile and did not display any observable toxicity (Yoo et al., 2021). NA49 induced HSP27 cross-linking and abnormal dimerization in L132 lung epithelial cells (Figure 1B). The IC_50_ value of NA49 in cellular cytotoxicity was similar to that of J2 in L132 cells (J2; 17.8 ± 0.75 μM and NA49; 11.4 ± 0.42 μM) (Supplementary Figure S1A). IR and TGFβ-mediated EMT, which was represented by increased expression of Twist, Snail, Fibronectin and α-SMA, as well as decreased expression of Zo-1, was dramatically reversed by 3 h pretreatment with NA49 (Figures 1C, D). Because J2 was previously reported to inhibit IR-induced morphologic changes, as well as the expression of IL-6, IL-1β and Twist, which are involved in inhibition of IR-mediated PF (Kim et al., 2019), we tested these phenomena using NA49 after treatment with IR or TGFβ. The L132 cells had a round or polygonal shape and had very close cell–cell contact, while the treatment with IR or TGFβ caused the cells to undergo a transformation, adopting a spindle-shaped morphology reminiscent of cellular tight junctions. However, cells pretreated with NA49 showed inhibition of these IR or TGFβ-induced morphologic features, and the restoration efficiency of morphology was similar in NA49 and J2 (Figure 1E). We next examined whether NA49 treatment modulated IR or TGFβ-induced *twist1, il-6,* and *il-1β* mRNAs. Quantitative RT-PCR analysis of L132 cells demonstrated that the upregulation of *twist1, il-6*, and *il-1β* genes induced by IR or TGFβ was effectively suppressed by NA49. The suppressive effect of NA49 was comparable to or even more potent than that of J2 (Figure 1F).

### NA49 inhibited BLM-induced PF in mice

**FIGURE 2:**
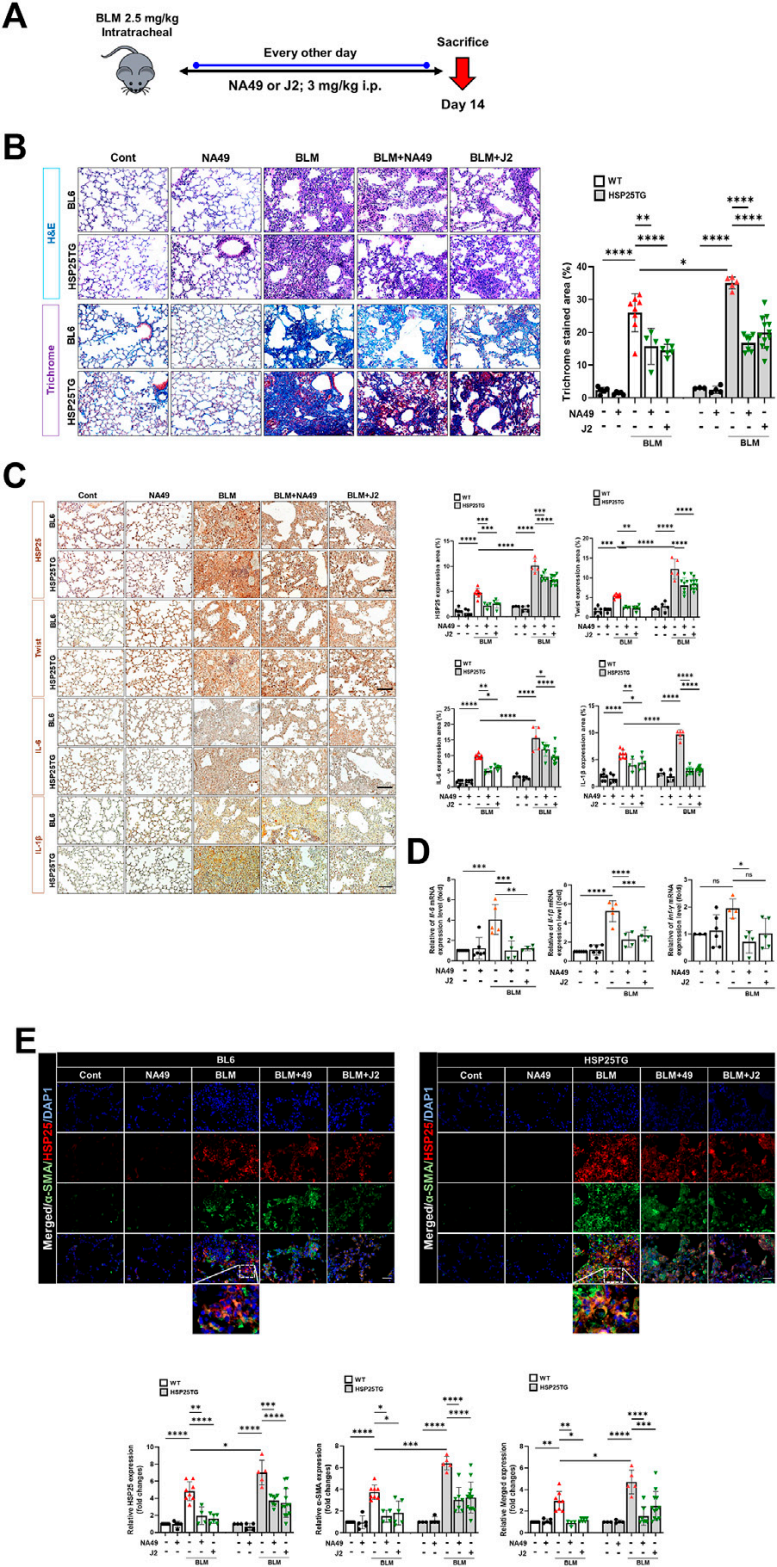
HSP27 cross linker NA49 attenuates BLM-induced in PF. NA49 and J2 (3 mg/kg) were intraperitoneally administered every other day after BLM treatment and lungs of BL6 and HSP25TG mice were harvested on day 14. **(A)** *In vivo* scheme of BLM-induced PF. **(B)** Represented images showed H&E and MT staining of lung tissue and **(C)** Immunostaining of HSP25, Twist, IL-6, and IL-1β. **(D)** BL6 mice were injected with BLM and additionally administered NA49 or J2. After 6 weeks of BLM treatment, lungs were analyzed for quantitative RT-PCR analysis. **(E)** Immunofluorescence (IF) staining was performed to identify the area co-stained with HSP25 (red) and α-SMA (green). Magnification, ×400. Quantitative analysis of staining intensity using ImageJ software. Data are expressed as mean ± SEM (n ≥ 3 per groups). **(B,C)** Bar plot of Trichrome area and protein area (%). ^*^ *p* < 0.05^**^ *p* < 0.01 ^***^ *p* < 0.001, and ^****^ *p* < 0.0001 (*p*-valued by ANOVA). Magnification; ×200 (H&E and MT), ×400 (IF); Scale bar: 50 µm.

To elucidate whether NA49 can inhibit BLM-induced PF in mice, we compared the alterations in lung surface morphology between the control group and the group treated with BLMs. We also investigated the effects of NA49 on BLM-induced PF in HSP25TG mice. J2 was treated to compare the effects of NA49 (Figure 2A). The H&E staining data indicated that intraperitoneal administration of NA49 resulted in less tissue injury compared to mice treated with BLM alone. In the BLM group, there was a notable increase in alveolar infiltration of inflammatory cells and the formation of alveolar hyaline membranes, which was significantly higher compared to the control group. However, mice treated with NA49 showed a decrease in tissue damage. HSP25TG mice were also compared, and NA49 treatment reduced the inflammation scores. MT staining revealed a significant increase in collagen accumulation in the BLM-exposed group compared to the control group. However, treatment with NA49 effectively reversed the deposition of collagen. Two weeks following BLM treatment, the lungs exhibited ground-glass opacities and consolidation. In contrast, NA49-treated mice showed a reduction in these effects. The recovery effects for PF were similar between J2 and NA49 treated mice. We also compared the inhibitory effects of collagen deposition in HSP25TG mice. TG mice showed more aggravated lung injury grossly and histologically after BLM treatment than BL6 mice, which was also attenuated by NA49 treatment. Similar to BL6 mice, the inhibitory effects were similar in J2 or NA49 treated TG mice (Figure 2B). Immunohistochemical analysis revealed that HSP25 protein expression in lung tissues increased as PF progressed. The levels of IL-6, IL-1β and Twist, which were identified as genes modulated by J2 in BLM- or IR-induced PF (Kim et al., 2019) and were more prominent in HSP25TG mice, were also inhibited by NA49 treatment in both normal BL6 and HSP25TG mice with similar potency between J2 and NA49 (Figure 2C). The mRNA levels of *il-6, il-1β* and *twist* which were increased in BLM-induced lung tissues, were also declined by treatment of J2 or NA49 (Figure 2D). The co-localization of HSP25 and α-SMA was investigated, and the double-stained HSP27+/α-SMA+ area was increased in the BLM-induced PF model, and this portion was inhibited by the treatment with NA49. The amount of double-stained HSP27+/α-SMA+ after treatment with BLM was greater in HSP25TG mice than in normal BL6 mice, and NA49 also significantly inhibited this area with similar potency J2 (Figure 2E).

### NA49 inhibited IR-induced PF in mice

**FIGURE 3:**
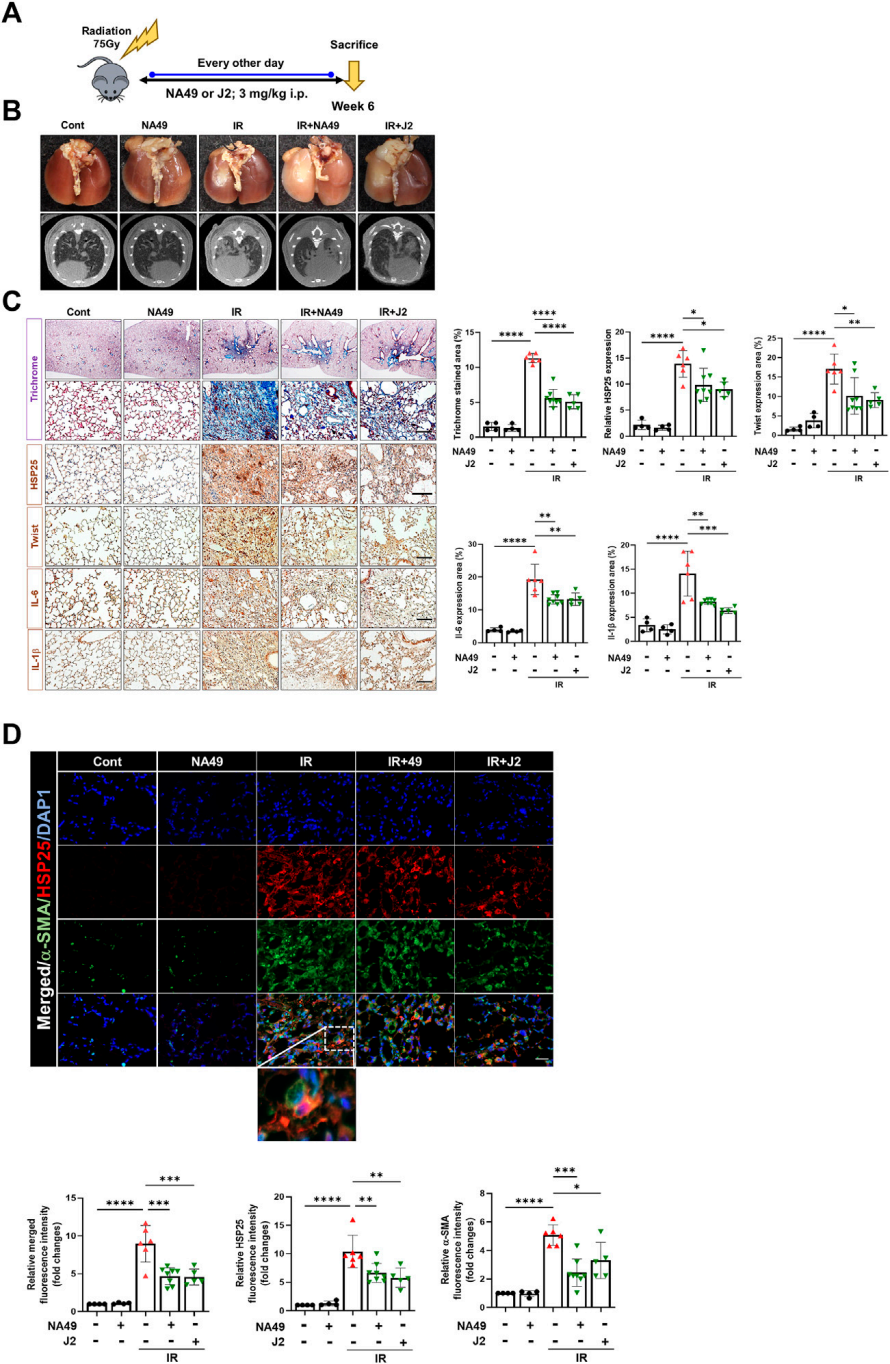
HSP25 cross linker NA49 attenuates IR-induced PF. NA49 and J2 (3 mg/kg) were intraperitoneally administered every other day after high dose radiation (75 Gy) and lungs were harvested on week 6. **(A)** *In vivo* scheme of IR-induced PF. **(B)** Lung was photographed after complete fixation, horizontal images acquired at 6 weeks after IR. **(C)** Represented images showed MT staining of lung tissue and immunostaining of HSP25, Twist, IL-6, and IL-1β. Bar plot of Trichrome area and protein area (%). **(D)** Immunofluorescence staining was performed to identify the area co-stained with HSP25 (red) and α-SMA (green). Magnification, ×400. Quantitative analysis of staining intensity using ImageJ software. Data are expressed as mean ± SEM (n ≥ 3 per groups); ^*^ *p* < 0.05 ^**^, *p* < 0.01 ^***^ *p* < 0.001 and ^****^ *p* < 0.0001 (p-valued by ANOVA). Magnification; ×200 (H&E and MT), 400X (IF); Scale bar: 50 µm.

We also investigated NA49 effects using another PF model, the IR-induced PF model (Figure 3A). Irradiated mice displayed a notable white, ring-like appearance in their lungs, which was partially restored by the administration of NA49 treatment. Six weeks after IR, the typical micro-CT manifestations of lung injury were observed in the irradiated left lung. However, treatment with NA49 also mitigated these manifestations (Figure 3B). Infiltration of inflammatory cells in the alveolar region and the formation of hyaline membranes within the alveoli were significantly increased in the IR group compared to the control group. MT staining indicated that the IR group had much more collagen deposition than the control group, which was dramatically ameliorated by NA49 treatment. Immunohistochemical staining data also suggested that the increased expression of HSP27, Twist, IL-1β, and IL-6 was reduced by NA49 (Figure 3C). The co-localization of HSP25 and α-SMA was studied, and similar to the BLM-induced PF model, the double-stained HSP27+/-SMA+ region became more in the IR-induced PF model, and that region was considerably decreased by NA49 treatment (Figure 3D). The inhibition rate of PF by NA49 or J2 was almost similar.

### NA49 reduced lung inflammation

**FIGURE 4:**
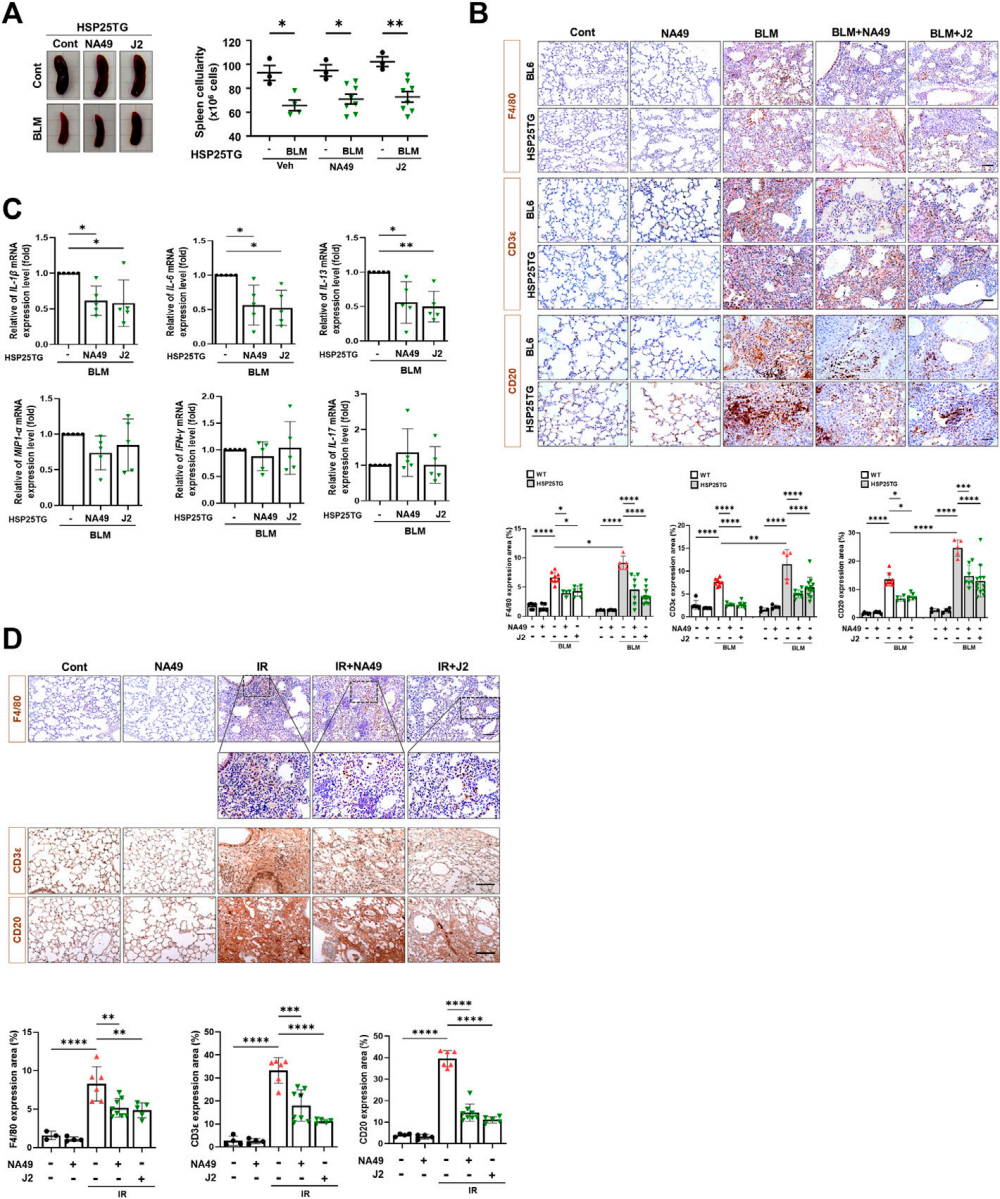
Attenuation of systemic and lung inflammation by NA49 in B6 and HSP25TG mice. **(A)** HSP25TG mice were injected with either PBS (veh, *n* = 3) or BLM together with Cont (*n* = 4), NA49 (*n* = 8) or J2 (*n* = 8). Spleen cellularity was determined by trypan blue exclusion assay. ^*^ *p* < 0.05. **(B)** Decreased pro-inflammatory cytokines by NA49 and J2 in BLM-treated HSP25TG mice. HSP25TG mice were injected with BLM and additionally administered Cont (*n* = 5), NA49 (*n* = 5) or J2 (*n* = 5) and spleen was obtained for the cytokine analysis. Relative cytokine gene expression was determined after normalization with actin level. ^*^ *p* < 0.05; ^**^ *p* < 0.005. Represented images showed immunostaining of CD3ε, CD20, and F4/80 in HSP25TG mice in BLM **(C)** or IR **(D)** induced fibrosis model (75 Gy). Quantitative analysis of staining intensity using ImageJ software. Bar plot of Protein area (%). Data are expressed as mean ± SEM (n ≥ 3 per groups); ^*^ *p* < 0.05 ^**^ *p* < 0.01 ^***^ *p* < 0.001 and ^****^ *p* < 0.0001 (*p*-valued by ANOVA). Magnification, ×200; Scale bar: 50 µm.

Since HSP25 induction in lung tissue aggravates IR-induced lung fibrosis, and the elevated infiltration of macrophages, B cells, and T cells was significantly reduced by J2 treatment in HSP25TG mice (Oh et al., 2021), in this study, we aimed to investigate the effects of NA49 on immune cells from splenocytes after treatment of BLM in HSP25TG mice, comparing its efficacy with that of J2. BLM injection caused splenopathy in HSP25TG mice, as evidenced by decreased splenic cellularity (Figure 4A). Treatment with NA49 had no effect on splenic cellularity of HSP25TG mice in the case of untreated BLM mice. Since fibrosis is triggered and promoted by inflammatory cytokines, such as macrophage-associated IL-1β, IL-6, and MIP1α, Th2-related IL-13, and Th17 cell-derived IL-17, we examined the effects of NA49 on the production of inflammatory cytokines. Quantitative RT-PCR analysis revealed that NA49 significantly reduced the expression of pro-inflammatory cytokines IL-1β, IL-6, and IL-13 in HSP25TG mice. However, NA49 did not have an effect on the expression of MIP1α, IFN-γ, and IL-17 (Figure 4B). Next, we conducted an investigation to assess the effects of HSP27 inhibitors on airway inflammation induced by IR and BLM in control B6 WT and HSP25TG mice. Moreover, consistent with expectations, both BLM and IR led to an elevation in immune cell infiltration in the lungs of WT mice. Moreover, immune cell infiltration was notably intensified in the lungs of HSP25TG mice. NA49 injection significantly reduced the infiltration of macrophages (F4/80 positive), T cells (CD3ε positive), and B cells (CD20 positive) stimulated by BLM or IR treatment in both WT and HSP25TG mice (Figures 4C, D). These findings indicate that NA49 has immunomodulatory action similar to J2 and may similarly contribute to the prevention of PF through the reduction of pro-inflammatory cytokines, which is mediated via immune cell regulation.

### NA49 inhibited HSP27-mediated NFkB activation

**FIGURE 5:**
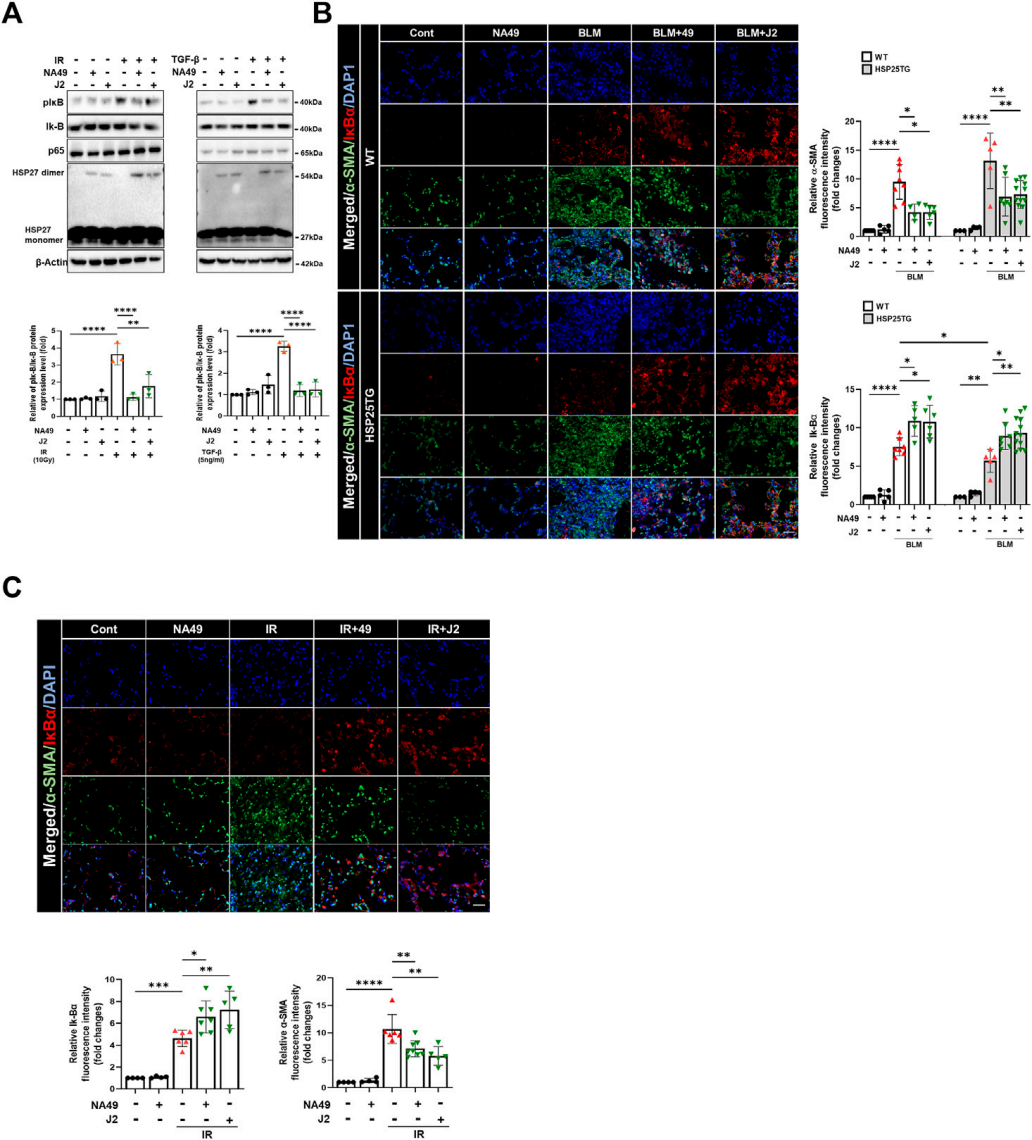
NA49 inhibits NFκB pathway in lung cell line. **(A)** Using L132 cells, the protein expression levels after 3 h treatment of 10 Gy irradiation (IR) or 5 ng/mL TGFβ with or without NA49 and J2 (0.5 µM) pretreatment were verified by immunoblot analysis. The intensity band was quantified by ImageJ software, and the data were normalized to β-actin. **(B, C)** Immunofluorescence staining was performed to identify the area co-stained with IkB (red) and α-SMA (green). Magnification, ×400; Scale bar: 50 µm. Quantitative analysis of staining intensity using ImageJ software. Data are expressed as mean ± SEM (n ≥3 per groups); ^*^ *p* < 0.05 ^**^, *p* < 0.01 ^***^ *p* < 0.001 and ^****^ *p* < 0.0001 (*p*-valued by ANOVA).

A latest study revealed that focused irradiation upregulated *twist1, il-6,* and *il-1β* genes through NFkB activation via HSP27 overexpression, while J2 reversed these effects (Kim et al., 2019). Therefore, we investigated whether NA49 can also inhibit BLM- or IR-mediated NFkB activation, which is represented by the inhibition of IkBα phosphorylation. After 3 h of IR or TGFβ1 treatment in L132 cells, NA49 dramatically inhibited IkBα phosphorylation (Figure 5A). Furthermore, NFkB activation in the fibrotic region of the BLM or IR-induced PF mouse model, as reflected by IkBα degradation, was greater in HSP25TG mice, and these phenomena were reduced by NA49 treatment when detected by immunofluorescence analysis (Figure 5B), indicating that IkBα expression was lower in fibrotic lungs compared to control mice and NA49 treatment increased the intensity of IkBα fluorescence. NA49 also showed similar effects in the case of IR-induced PF model (Figure 5C). The inhibition rate of NFkB activation by NA49 or J2 was almost similar.

### NA49 showed less DNA stand break damage than J2

**FIGURE 6:**
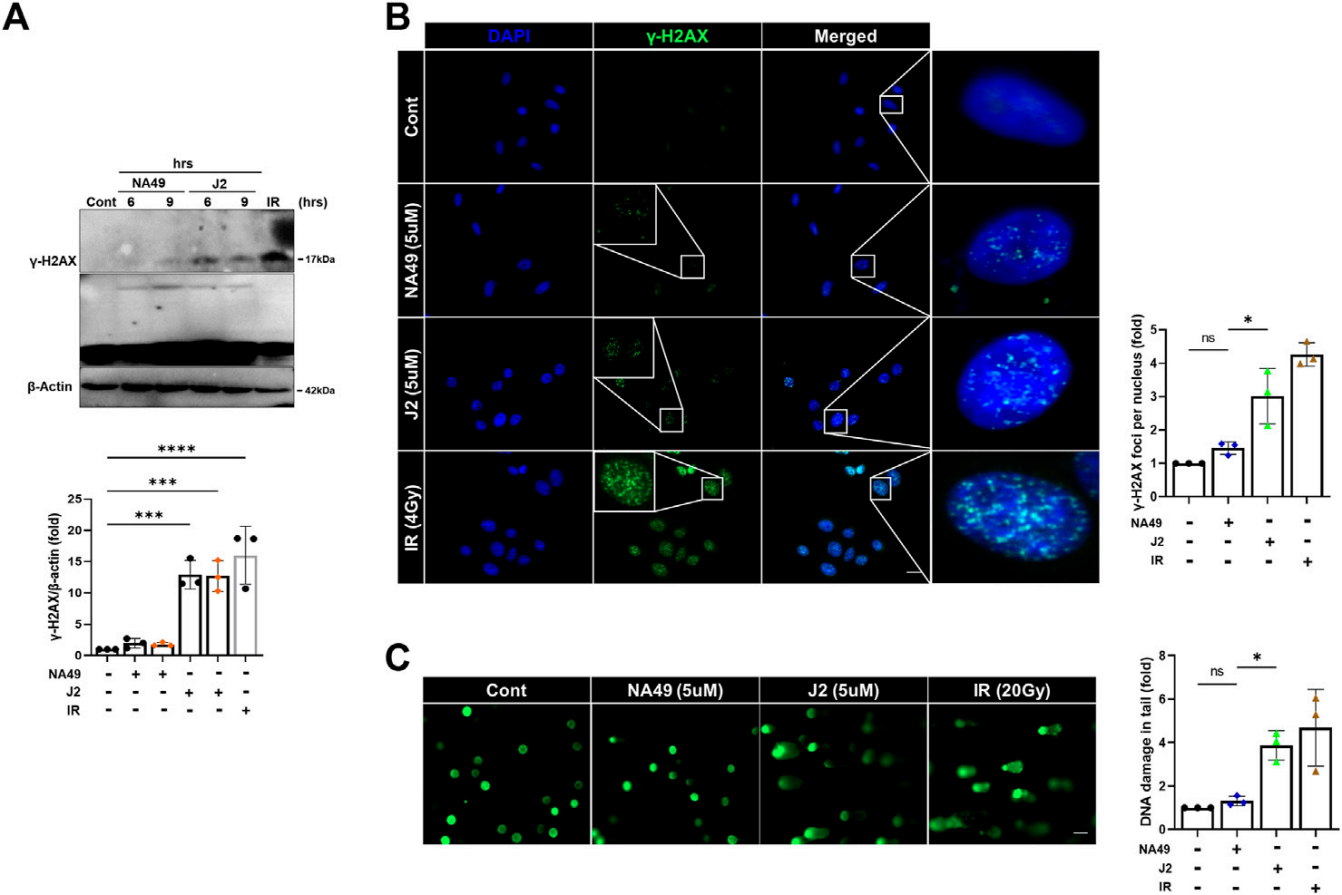
NA49 does not induce DNA damage toxicity, unlike J2. **(A)** L132 cells were treated with 6 and 9 h of 5 µM NA49 or J2 and protein expression was verified using immunoblots. IR (1 Gy) was used as a positive control. The intensity band was quantified by ImageJ software, and the data were normalized to β-actin. **(B)** L132 cells were treated with 5 µM NA49 or J2 for 1 h. Immunofluorescence staining was performed to identify the area co-stained with DAPI (blue) and γ-H2AX (green). IR 4 Gy was also applied. γ-H2AX foci number was quantified using ImageJ software. **(C)** L132 cells were treated with 5 µM NA49 or J2 for 24 h, and the alkaline comet assay was conducted, and representative photos are included. IR 20 Gy was used as a positive control. The olive tail moment was determined using Comet 5.5 software. The data represent the mean ± SD. Magnification: ×5; Scale bar: 50 µm. **(C)** Data are expressed as mean ±SEM; Scale bar: Magnification, ×400; Scale bar: 50 µm *p < 0.05 **p < 0.01 ***p < 0.001 and ****p < 0.0001 (*p*-valued by ANOVA).

Due to the limited solubility and short *in vivo* circulation time of J2, NA49 was developed as a more druggable compound. NA49 exhibits a superior pharmacokinetic profile compared to J2, showing improved characteristics such as enhanced bioavailability, longer circulation time, and negligible toxicity (Yoo et al., 2021). Furthermore, the exposure to J2 at a concentration of 40 µg/plate for 48 h led to a significant increase in bacterial reverse mutation in the Ames test, utilizing *Salmonella typhimurium* strains TA98 and TA100, compared to the control vehicle group. In contrast, treatment with 200 µg/plate of NA49 for 48 h did not show a significant increase in the number of relevant colonies (Yoo et al., 2021). Therefore, to elucidate whether NA49 or J2 also differentially affect DNA strand break damage, we examined γ-H2AX expression and comet tail moments. J2 increased γ-H2AX expression at a concentration of 5 μM; however, in the case of NA49, γ-H2AX induction was not observed at the same concentration (Figure 6A). Immunofluorescence of γ-H2AX foci, an indicator of the DNA strand breaks, also suggested that NA49 only slightly, but no significantly induced γ-H2AX foci in the cells, while J2 dramatically induced γ-H2AX foci. The nucleus was stained with DAPI (blue) (Figure 6B). Similarly, alkaline comet tail moments were significantly induced by J2 treatment; however, NA49 did not show any significant induction of comet tail moments (Figure 6C). IR exposure to the cells was used as a positive control.

## Discussion

In this study, we proposed NA49, a small molecule HSP27 inhibitor, as a potential therapeutic agent for PF. NA49, which shares a structural similarity with the chromenone compound J2, exhibited comparable levels of altered HSP27 cross-linking as J2. However, NA49 demonstrated improved druggability and comparable potency for PF, along with superior toxicity profiles, making it a promising candidate for further development. Moreover, unlike J2, NA49 did not show any DNA strand break damage at the same concentration.

Previously, we have demonstrated the novel mechanisms of HSP27 in the development of PF and proposed HSP27 as a potential therapeutic target for treating PF. We observed HSP27 upregulation during the development of PF. Additional evidence of HSP27 overexpression in PF comes from proteomics studies, which have shown the upregulation of HSP27 in lung fibroblast cell lines following treatment with TGFβ1 (Malmström et al., 2004) and in IPF lung tissues (Kim et al., 2019). Recent studies have reported the successful mitigation of BLM-induced PF in mice through the administration of HSP27 siRNAs via the airway by downregulation of myofibroblast-related proteins, including fibronectin and type 1 collagen (Park et al., 2016). In addition, the effectiveness of HSP27 antisense oligonucleotide in suppressing subpleural fibrosis induced by adenovirus-expressing TGFβ1 in rats has been demonstrated. Furthermore, HSP27 has been found to prevent Snail degradation through the proteasomal system (Hagiwara et al., 2007). Elevated HSP27 levels have been found to exacerbate NF-κB signaling pathways, leading to increased EMT. However, the use of HSP27 inhibitors, such as J2, has been shown to effectively prevent PF during the inflammation or EMT. We also looked at the impact of delaying J2 medication till late in the fibrosis process (Jeon et al., 2023). Therefore, HSP27 inhibition at EMT stage is likely to give a useful medical care option for PF, which presently has few viable treatment alternatives.

PF is a progressive fibrotic disease with pathophysiological characteristics of TGFβ and reactive oxygen species-induced excessive EMT and ECM deposition. Several research studies have highlighted the involvement of various types of cells, such as epithelial cells, macrophages, and fibroblasts, in the progression of PF (Wynn, 2011). According to current understanding, it is widely believed that epithelial cells contribute to the formation of myofibroblasts through the process of EMT (Radisky et al., 2007). EMT has the capability to trigger transcription factors, release cell surface proteins and cytokines associated with fibrosis, and enhance the accumulation of EMT (Di Gregorio et al., 2020). The conversion of fibroblasts into myofibroblasts is a key characteristic of fibrotic conditions, resulting in the excessive production and accumulation of ECM components such as collagen, fibronectin, and elastin (Sun, 2021). Therefore, the inhibition of EMT, inflammatory cascade activation, and fibroblast stimulation emerge as potential strategies for mitigating PF (Kendall and Feghali-Bostwick, 2014).

Another important inhibitory mechanism of HSP27 on IR- or BLM-induced PF was due to the modulation of inflammatory immune systems. HSP27 is implicated in the infiltration and activation of macrophages, B cells, and T cells, which potentiated the inflammatory cytokine production (Oh et al., 2021). Although relationship between immune cell modulation and EMT process by HSP27 is not known exactly, it is clear that PF is improved by various complex mechanisms by HSP27 inhibition by a drug like compound, NA49.

Despite the fact that presently authorized IPF treatments, such as PFD and NTD, are clinically accessible, there are certain limits in terms of side effects and efficacy. As a result, an alternative method is required to meet the unmet therapeutic need for PF, and the discovery and clinical use of novel HSP27 inhibitors will provide an additional therapeutic option for overcoming IPF. However, unlike HSP90 and HSP70, HSP27 does not have an active site or an ATP-binding pocket. As a result, only two HSP27 inhibitors are currently in clinical studies. Nevertheless, the restricted intracellular delivery of OGX427 is attributed to its small molecular size and the lack of a mechanism targeting HSP27 specifically in the case of RP101 (Bartis et al., 2014). Apart from RP101, there have been no other small molecules developed as inhibitors of HSP27, and the existing clinical trial data for RP101 is not promising. While approved treatments like PFD and NTD are currently available for PF, there is a pressing need for an alternative approach to address the unmet therapeutic requirements of PF.

**FIGURE 7:**
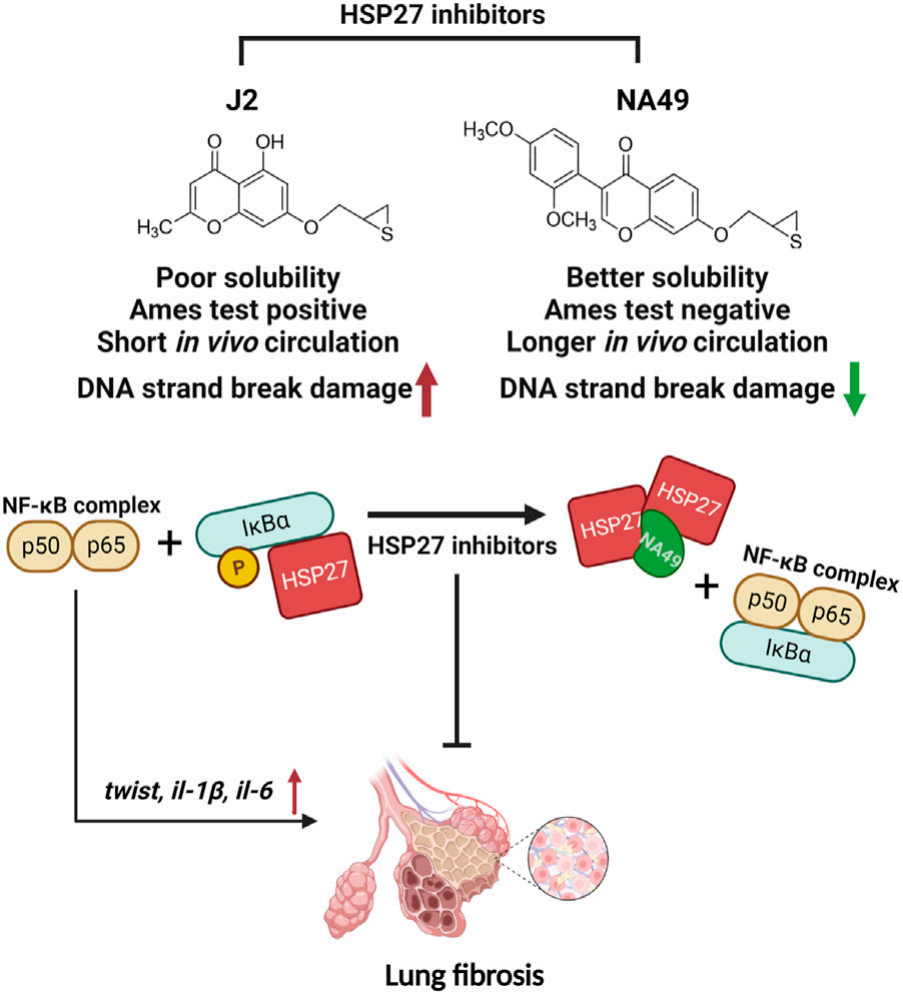
The graphic illustration of the pathway of NA49 protecting against PF. NA49, a small molecule HSP27 inhibitor, induces the abnormal dimerization of HSP27 and maintains the binding of NF-kB and IkBα, thereby effectively inhibiting the transcriptional activity of NF-kB, which provides the evidence for the alleviation of PF. NA49 exhibiting higher solubility than J2 and negative results in the AMES test while also displaying improved pharmacokinetic parameters, shows relatively lower DNA strand break damage. Figure 7 was created with BioRender.com accessed on 8 June 2023.

Previously, small molecule HSP27 inhibitors, such as ZER, SW15, and J2, have been shown to induce the formation of abnormal HSP27 dimers, were developed, and J2 showed inhibition of PF in various mouse models (Kim et al., 2019; Yoo et al., 2021; Jeon et al., 2023). A drug’s chemical structure impacts its physicochemical properties of a drug are directly influenced by its chemical structure, and these properties, in turn, impact the drug’s ADME/Tox characteristics, ultimately determining its pharmacological effectiveness. Therefore, our focus was on creating drug-like molecules with potentially favorable ADME/Tox properties. In pursuit of this objective, we conducted studies using J2, an effective HSP27 cross linker that is a synthetic chromenone derivative. However, J2 exhibits limitations such as poor solubility and a short *in vivo* circulation time, and positive Ames test results, we aimed to identify more druggable compounds that exhibit similar or superior efficacy to J2. Among these compounds, NA49, which is also a chromenone compound, demonstrated the desired effects. NA49 exhibited comparable levels of HSP27 cross-linking to J2. NA49 demonstrates lower toxicity and a superior pharmacokinetic profile in comparison to J2, even without mutation toxicity (Yoo et al., 2021), and showed similar effects on PF inhibition induced by BLM or IR. Moreover, NA49 did not show a DNA strand break in normal lung epithelial cells, unlike J2 (Figure 7).

In summary, our data demonstrate that a druggable HSP27 inhibitor with minimal toxicity, NA49 efficiently ameliorates PF progression by suppressing EMT and inflammation. Our study indicates that NA49 may serve as a clinically available drug for the treatment of PF.

## Data Availability

The original contributions presented in the study are included in the article/Supplementary Material, further inquiries can be directed to the corresponding author.
