# Supplementary material for: Drug like HSP27 cross linkers with chromenone structure ameliorates pulmonary fibrosis
Source: Front Pharmacol. 2023 Jul 4;14:1203033. doi: 10.3389/fphar.2023.1203033 (PMC10352808; doi:10.3389/fphar.2023.1203033)
Supplement: Supplementary file 1 [file Table1.DOCX]

**Supplementary Information**

**Materials and methods**

**Cell Culture**

L132 (human normal lung epithelial cell line) were obtained from the ATCC and cultured in RPMI or DMEM (Gibco) supplemented with 10% FBS (Gibco) in a 37 C incubator with 5% CO_2_. Cell lines were tested by BioMycoX Mycoplasma PCR Detection Kit (JCBIO Co., Ltd) to ensure that they were mycoplasma-free.

**Preparation of lung tissues for immunohistochemistry and Immunofluorescence staining**

Lung tissue was isolated from IR or BLM-treated mice, fixed in 10% (v/v) neutral buffered formalin, and used for paraffin embedding. Paraffin-embedded sections were deparaffinized and stained with hematoxylin and eosin (H&E, Sigma-Aldrich), a Masson’s trichrome stain kit (Sigma-Aldrich), immunohistochemistry, and immunofluorescence staining.

**Immunohistochemistry (IHC)**

IHC staining was carried out using antibodies at 4℃ overnight. Slides were then incubated with Avidin–Biotin peroxidase complex (ABC kit, Vector Laboratories) and developed using 3, 30-diaminobenzidine tetrachloride (DAB; Thermo Fischer Scientific). Images were obtained from a microscope (Zeiss, Oberkochen, Germany) equipped. To evaluate the positive index on H&E staining and immunohistochemistry slides, 10 different regions of each mouse lung were quantitated with Image J software using standard algorithms (National Institutes of Health, Bethesda, MD, USA. The specific primers used are listed in Supplementary Table S3.

**Immunofluorescence (IF)**

For immunoﬂuorescence staining, cells or tissue sections stained with antibodies (Supplementary Table S3) were incubated with appropriate ﬂuorescent secondary antibodies and counterstained with 4, 6-diamidino-2-phenylindole dihydrochloride (DAPI). Images were viewed under a confocal microscope, Zeiss.

| **Antibody** | **Source** | **Host Species** | **Working Concentration** |
| --- | --- | --- | --- |
| Zo-1 | Thermo Fisher Scientiﬁc, 40-2200 | Rabbit | 1:1000 |
| Twist | Santa Cruz Biotechnology, sc-15393 | Mouse | 1:1000 |
| Snail | Cell signaling Technology, CST3895S | Mouse | 1:1000 |
| Fibronectin | Becton-Dickinson Laboratories, BD610077 | Mouse | 1:1000 |
| α-SMA | Sigma, A5228 | Mouse | 1:1000 |
| HSP27 | Santa Cruz Biotechnology, sc-13132 | Mouse | 1:1000 |
| β-actin | Santa Cruz Biotechnology, sc-47778 | Mouse | 1:1000 |

Supplementary Table S1. List of antibodies, their source and working dilutions in immunoblotting

| **Gene** | **Species** | **Primers 5’-3’** |
| --- | --- | --- |
| GAPDH | Human | F: TGT AGT TGA GGT CAA TGA AGG G  R: ACA TCG CTC AGA CAC CAT G |
| Twist | Human | F: GTC CGC AGT CTT ACG AGG AGC  R: GCT TGA GGG TCT GAA TCT TGC T |
| IL-1β | Human | F: TGA GCT CGC CAG TGA AAT GA  R: AAC ACG CAG GAC AGG TAC AG |
| IL-6 | Human | F: TTC GGT CCA GTT GCC TIC TC  R: CAG CTC TGG CTT GTT CCT CA |

Supplementary Table S2. List of primers in quantitative RT-PCR

| **Antibody** | **Staining** | **Source** | **Host Species** | **Working Concentration** |
| --- | --- | --- | --- | --- |
| Twist | IHC | GeneTex, GTX127310 | Rabbit | 1:100 |
| IL-1β |  | Abcam, ab9722 | Rabbit | 1:100 |
| IL-6 |  | Abcam, ab208113 | Rabbit | 1:100 |
| CD3ε |  | Abcam, ab167755 | Rabbit | 1:100 |
| CD20 |  | Santa Cruz Biotechnology,  sc-393894 | Mouse | 1:100 |
| F4/80 |  | Abcam, ab16911 | Rabbit | 1:100 |
| α-SMA | IF | Sigma, A5228 | Mouse | 1:200 |
| IκBα |  | Abcam, ab32518 | Rabbit | 1:100 |
| HSP27 |  | Santa Cruz Biotechnology, sc-13132 | Mouse | 1:1000 |
| Alexa488-conjugated phalloidin staining |  | Invitrogen, A12379 | | 1:200 |

Supplementary Table S3. List of antibodies, their source and working dilutions in immunohistochemistry and immunofluorescence.

**Supplementary Figure S1**: IC50 values after treatment of NA49 or J2 in L132 cell line. L132 cells were treated with different concentrations of NA49 or J2 for 24 h and cell viability was analyzed by MTT assay (mean±SD).
