# Supplementary figures and images for: Drug like HSP27 cross linkers with chromenone structure ameliorates pulmonary fibrosis
Source: Front Pharmacol. 2023 Jul 4;14:1203033. doi: 10.3389/fphar.2023.1203033 (PMC10352808; doi:10.3389/fphar.2023.1203033)

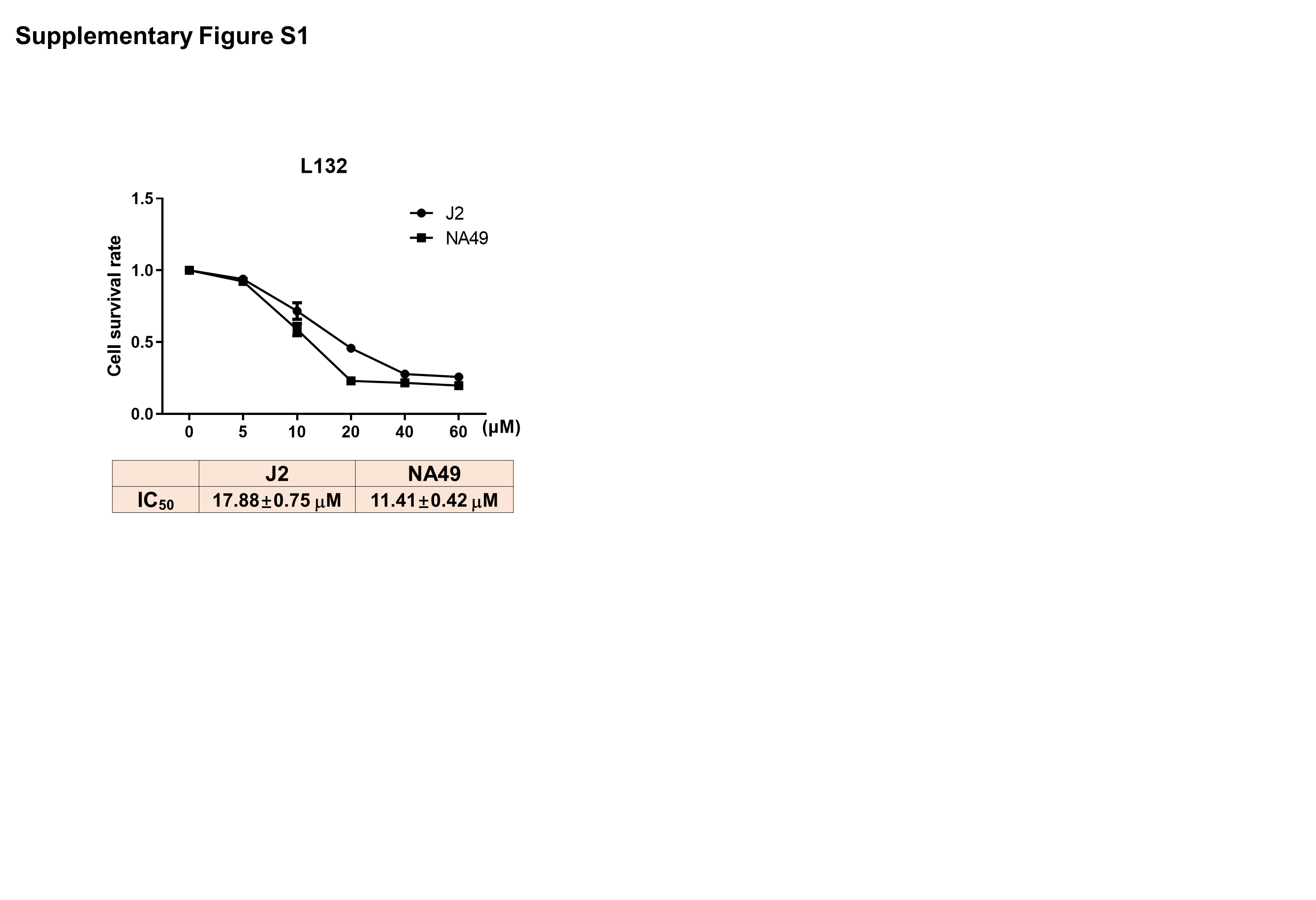

Supplement: Supplementary file 2 [file Image1.TIF]
